# A simple clinical model for planning transfusion quantities in heart surgery

**DOI:** 10.1186/1472-6947-11-44

**Published:** 2011-06-21

**Authors:** Felicetta Simeone, Federico Franchi, Gabriele Cevenini, Antonino Marullo, Vittorio Fossombroni, Sabino Scolletta, Bonizella Biagioli, Pierpaolo Giomarelli, Paolo Barbini

**Affiliations:** 1 Unit of Cardiothoracic Anaesthesia and Intensive Care, Azienda Ospedaliera Universitaria Senese, Siena, Italy; 2 Department of Surgery and Bioengineering, Università di Siena, Siena, Italy; 3 Unit of Immune Haematology and Transfusion Medicine, Azienda Ospedaliera Universitaria Senese, Siena, Italy

## Abstract

**Background:**

Patients undergoing heart surgery continue to be the largest demand on blood transfusions. The need for transfusion is based on the risk of complications due to poor cell oxygenation, however large transfusions are associated with increased morbidity and risk of mortality in heart surgery patients. The aim of this study was to identify preoperative and intraoperative risk factors for transfusion and create a reliable model for planning transfusion quantities in heart surgery procedures.

**Methods:**

We performed an observational study on 3315 consecutive patients who underwent cardiac surgery between January 2000 and December 2007. To estimate the number of packs of red blood cells (PRBC) transfused during heart surgery, we developed a multivariate regression model with discrete coefficients by selecting dummy variables as regressors in a stepwise manner. Model performance was assessed statistically by splitting cases into training and testing sets of the same size, and clinically by investigating the clinical course details of about one quarter of the patients in whom the difference between model estimates and actual number of PRBC transfused was higher than the root mean squared error.

**Results:**

Ten preoperative and intraoperative dichotomous variables were entered in the model. Approximating the regression coefficients to the nearest half unit, each dummy regressor equal to one gave a number of half PRBC. The model assigned 4 units for kidney failure requiring preoperative dialysis, 2.5 units for cardiogenic shock, 2 units for minimum hematocrit at cardiopulmonary bypass less than or equal to 20%, 1.5 units for emergency operation, 1 unit for preoperative hematocrit less than or equal to 40%, cardiopulmonary bypass time greater than 130 minutes and type of surgery different from isolated artery bypass grafting, and 0.5 units for urgent operation, age over 70 years and systemic arterial hypertension.

**Conclusions:**

The regression model proved reliable for quantitative planning of number of PRBC in patients undergoing heart surgery. Besides enabling more rational resource allocation of costly blood-conservation strategies and blood bank resources, the results indicated a strong association between some essential postoperative variables and differences between the model estimate and the actual number of packs transfused.

## Background

Despite published blood conservation and transfusion guidelines, transfusion practices in heart-surgery patients differ widely between physicians and institutions. For example, in Europe, packs of red blood cells (PRBC) are transfused in about half of all patients undergoing heart surgery, but their use varies from 8% to 90% depending on the institution [1]. A minority of patients (from 15% to 20%) need more than 80% of the blood products transfused during the operation [2].

Although blood transfusion is an essential therapy during surgical procedures, better quantification and limitation of the need for transfusions may improve clinical outcome [3]. It is difficult to define the advantages of blood transfusion, but enhanced oxygen-carrying capacity, improved hemostasis and cardiac function volume support are three important aspects [4,5]. However transfusion of blood packs has been more and more recognized as a risk factor for adverse outcome after heart surgery and unnecessary transfusions have been associated with increased morbidity and additional indirect hospitalization costs [6]. The Task Force on Blood Component Therapy of the American Society of Anesthesiologists developed a consensus statement suggesting that "red blood cell transfusions should not be dictated by a single hemoglobin trigger but instead should be based on the patient's risk of developing complications of inadequate oxygenation" [7].

Previous studies aimed at identifying a set of preoperative variables associated with need for blood transfusion in heart surgery patients [8-11]. In particular, Alghamdi and co-workers used a logistic regression approach to define an index based on eight preoperative variables [9]. The index was called Transfusion Risk Understanding Scoring Tool (TRUST). Karkouti and co-workers analysed data from heart surgery patients at seven Canadian hospitals to determine interhospital variation and predictability of large-volume transfusions [10]. They found interhospital variation that could not be explained by patient - or surgery-related factors. Ranucci and co-workers proposed a simple score, named Transfusion Risk and Clinical Knowledge (TRACK) [11]. This score only uses five preoperative variables to predict transfusion rate in heart surgery. Despite remarkable differences in transfusion practices and heart surgery procedures, these studies confirm the interest in developing protocols of blood conservation based on quantitative models obtained from available evidence.

Analysing a set of preoperative and intraoperative variables associated with transfusions in patients undergoing isolated coronary artery bypass grafting (CABG), isolated valve, or combined procedures (CABG plus valve), we propose a simple model, which does not require computers, to estimate the need for PRBC of new cases in clinical practice. This tool may help in the management of critical patients, when much time and attention is dedicated to medical and pharmacological care, because blood conservation can be most productive for high-risk subjects. The clinical course of patients showing the highest differences between actual and model-estimated number of blood packs was also analyzed for potential model weaknesses and to understand the reasons for significant discrepancies between model estimates and medical decisions.

## Methods

### Patient set and acquired variables

For the present observational study, 3315 consecutive patients between January 2000 and December 2007 were entered in a prospectively collected database and analyzed retrospectively. They underwent isolated CABG, single valve, or combined procedures at the Cardiac Surgery Unit of "Santa Maria alle Scotte" University Hospital, Siena, Italy. Exclusion criteria included age less than 18 years, operation without cardiopulmonary bypass (CPB), heart or heart-lung transplant and aortic dissection. Patients were assigned at random to two sets of equal size: a training set used to design the model and a testing set used to verify model performance on new data.

Data was obtained from the hospital database by retrieving baseline demographic and clinical information collected prospectively by clinical coordinators and entered in the database by trained data-management personnel. The study was undertaken after the approval of the Ethics Committee (Comitato etico locale e comitato etico per la sperimentazione clinica dei medicinali) of Siena University Hospital. Due to the retrospective nature of the study, the need for informed consent was waived.

Blood transfusion was quantified as the number of PRBC administered in the intensive care unit. The preoperative and intraoperative variables listed in Table 1 were considered a likely independent-variable set for planning transfusion quantities in major heart surgery procedures.

**Table 1:** Descriptive statistics of preoperative and intraoperative variables analyzed in the whole sample (3315 patients)

Variables	Frequency count and percentage
Sex (female)	1093 (33.0%)

Age > 70 years	1549 (46.7%)

Body surface area > 1.8 m^2^	1329 (40.1%)

Surgical procedure different from isolated coronary artery bypass graft	1559 (47.0%)

Previous cardiovascular surgery	147 (4.4%)

Emergency	67 (2.0%)

Non-routine procedure (urgency)	287 (8.7%)

Preoperative dialysis	21 (0.6%)

Systemic arterial hypertension	2227 (67.2%)

Diabetes requiring medical treatment	780 (23.5%)

Unstable angina	695 (21.0%)

Active endocarditis	16 (0.5%)

Recent myocardial infarction (< 7 days)	98 (3.0%)

Cardiogenic shock	32 (1.0%)

Chronic obstructive pulmonary disease	228 (6.9%)

Previous cerebrovascular events	165 (5.0%)

Admission hematocrit ≤ 40%	1973 (59.5%)

Antiplatelet therapy	1469 (44.3%)

Dicoumarole therapy	136 (4.1%)

Heparin therapy	1225 (37.0%)

Intraortic balloon pump insertion	83 (2.5%)

Serum creatinine > 1.2 mg/dl	599 (18.1%)

Cardiopulmonary bypass time > 130 minutes	1351 (40.8%)

Aortic clamping time > 90 minutes	1445 (43.6%)

Minimum hematocrit during cardiopulmonary bypass ≤ 20%	533 (16.1%)

Minimum temperature ≤ 32 °C	1639 (49.4%)

A set of postoperative variables (Table 2) was also analysed for a clinical interpretation of model performance. Morbidity outcome was associated with patients developing at least one cardiovascular, respiratory, neurological, renal, infectious or hemorrhagic complication [12]. Mortality was defined as in-hospital death.

**Table 2:** Descriptive statistics of some essential postoperative variables in the whole sample (3315 patients)

Variables	Frequency count and percentage
Morbidity	1273 (38.4%)

Reoperation for bleeding	190 (5.7%)

Lung dysfunction	516 (15.6%)

Low cardiac output	442 (13.3%)

Cardiac arrhythmia	294 (8.9%)

Coma	35 (1.1%)

Stroke	33 (1.0%)

Acute kidney failure	71 (2.1%)

Kidney dysfunction	140 (4.2%)

Sepsis	26 (0.8%)

Pneumonia	51 (1.5%)

Sternal wound infection	17 (0.5%)

Death	58 (1.7%)

Mechanical ventilation > 1 day	422 (12.7%)

Intensive care > 5 days	391 (11.8%)

### Clinical management

A broad-based blood conservation strategy was practiced in all patients, including:

• preoperative optimization of hemoglobin;

• intraoperative isovolemic hemodilution;

• autotransfusion;

• anemia tolerance (Hb < 7 g/dl);

• ultrafiltration during CPB for severe hemodilutional anemia or diagnosis of renal failure;

• on-site coagulation monitoring (using thromboelastography or activated clotting time);

• targeted pharmacotherapy (antifibrinolytic agents).

Patients were operated under moderate hypothermia (34°C) and α-stat acid-base management. Roller or centrifugal pumps were used with standard or biocompatible circuits (heparin or phosphorylcholine treated) and hollow-fibre oxygenators; the CPB circuit was primed with crystalloid or colloid solutions at variable volumes ranging from 900 to 1500 ml. Addition of PRBC to the CPB machine prime was considered (though not routinely given) if the calculated dilutional hematocrit was less than 25%. Anticoagulation was established with heparin to reach and maintain a target activated clotting time of 480 s. In all patients, heparin was reversed by adequate doses of protamine sulphate at the end of CPB. Pump flow was set between 2.0 and 2.8 l/(min m^2^), according to core temperature. During surgery with conventional open CPB circuits, blood was taken from the surgical field, collected in a reservoir, processed and re-infused into the patient. After the operation and during the first period of intensive care unit stay, mediastinal blood collected in a reservoir was not re-infused. Although no rigid criterion or hemoglobin value was adopted for transfusion, PRBC were not routinely considered until serum Hb was less than 7 g/dl, unless there was evidence of ongoing blood loss or the patient was clinically considered at risk of poor oxygenation. This last determination included patients with signs of poor tissue perfusion (lactate level < 2 mmol/l, mixed venous oxygen saturation < 65%, urine output < 0.5 ml/kg/h), significant hemodynamic instability requiring two or more inotropic agents, utilization of intraaortic balloon pump and multiple organ dysfunction. Fresh-frozen plasma was used in cases of postoperative bleeding and impaired coagulation factors (International Normalized Ratio > 1.5, i.e. thromboelastographic reaction time > 1.5 times the standard time). Platelet concentrates were administered in cases of postoperative bleeding associated with thromboelastographic maximal amplitude < 41 mm or low postoperative platelet count (< 50,000 cells/l).

### Model design and validation

A linear regression model was designed to assess the appropriate number of PRBC from training data. This type of model is quite flexible and categorical variables can be used as independent variables (regressors) without much difficulty. The simplest and most common way of creating variables to represent categories is known as *dummy variable analysis *[13]. It enabled us to divide patients into subgroups and to condense a considerable amount of information in a single equation.

To design a dummy-variable linear-regression model, it is necessary to create a series of binary (i.e. dummy) variables that identify whether or not an observation belongs to a specific category or group. Binary variables can only be coded as *one *or *zero*. If an observation is classified as a member of a particular category, then it is coded one on the dummy variable representing that category. Otherwise, the observation is coded zero. If a model only contains dummy variables, it is equivalent to analysis of variance. Regression with only dummy variables representing a single qualitative variable is equivalent to one-way analysis of variance.

Our model to evaluate number of PRBC contained only dummy regressors accounting for different categorical variables. To do this, all continuous independent variables were first dichotomized and then made binary by selecting suitable cut-off points related to intensive care unit morbidity [12], so that a dummy regressor was set equal to zero if the corresponding categorical risk factor was absent and to one if it was present. Since patients with low risk factors received a number of PRBC not significantly different from 0, a regression without an intercept was used, thus assuming that model output was zero when all dummy variables were zero.

A stepwise procedure was used to select an optimal subset of dummy regressors. Although stepwise methods may overfit the data, they are usually employed to reduce the number of potentially significant variables and increase model generalization, i.e. the model's ability to maintain performance on cases not used for model design. Stepwise regression is typically an automated process of building a model by successively adding or removing variables based solely on the *F*-statistics of their estimated coefficients. At each step, the process performs the following calculations: for each variable currently in the model, it computes the *F*-statistic for its estimated coefficient and reports this as its *F-to-remove *statistic; for each variable not in the model, it computes the *F*-statistic that its coefficient would have if it was the next variable added and reports this as its *F-to-enter *statistic. Then it enters the variable with the highest *F-to-enter *statistic, or removes the variable with the lowest *F-to-remove *statistic, according to specified control parameters. The process stops when no variable satisfies the criteria for inclusion or removal. In the present paper probability levels of 0.05 and 0.10 were set for *F-to-enter *and *F-to-remove *statistics, respectively.

Once the regression model was obtained, the regression coefficients were rounded to the nearest half unit, so that each dummy regressor equal to 1 simply corresponded to an integer number of half PRBC. Of course, for applicative purposes, the grand total of the model-assessed half packs was then rounded to the next superior integer of PRBC.

Model fit was evaluated by calculating the root mean square error (RMSE) representing the average difference between model-estimated and actual numbers of PRBC.

Since blood packs may sometimes be prescribed for clinical situations not necessarily related to patient preoperative or intraoperative condition, or subjectively by different operators, model accuracy was also evaluated, analyzing in detail the clinical course of all patients in cases where the absolute difference between the number of packs transfused and the number estimated by the model was greater than RMSE. This enabled remarkable differences to be interpreted clinically and model restrictions to be made for proper application. In particular, we divided model results into three categories: well transfused patients, where the absolute value of the model error was less than (or equal to) RMSE; less transfused patients, where the difference between the model estimate and the actual number of packs transfused was greater than RMSE; over-transfused patients, where the difference between the actual number of packs transfused and the model estimate was greater than RMSE.

The Mann-Whitney test was performed to compare transfused quantities between normal and morbid patients, considering actual and model-estimated number of PRBC. The Wilcoxon test for paired samples was also used to test differences between actual and model-estimated numbers of PRBC, taking normal and morbid patient groups separately.

The statistical association between percentage morbidity and the three groups of model agreement (or disagreement) was evaluated applying the chi-square test to contingency table. Pairwise group comparisons were also carried out by the Fisher exact test for analysis of 2 × 2 subtables. Fisher exact test was also used to verify frequency differences between training and testing data for model-selected dummy variables.

A p-value less than 0.05 was considered statistically significant for all statistical tests. All computations were done using SPSS and MATLAB code.

## Results

Essential descriptive statistics of the sample are summarized in Tables 1, 2 and 3. Patients underwent the following surgical procedures: 1756 (53%) isolated coronary artery bypass graft surgery, 1006 (30%) single-valve procedures (repair or replacement) and 553 (17%) other types of procedures, primarily combined coronary artery bypass graft surgery and valve surgery or aortic surgery.

**Table 3:** Patient numbers and related percentages by surgical procedure with respect to the whole sample of 3315 patients

Surgery procedure	Patient number	Transfused-patient number	Mean value ± SD of PRBC
CABG	1756 (53.0%)	1092 (62.3%)	2.0 ± 2.7

Valve	1006 (30.3%)	677 (67.3%)	2.6 ± 3.2

Other procedures	553 (16.7%)	439 (79.4%)	3.9 ± 3.5

The number and percentage of transfused patients are also reported for each surgical procedure together with the mean number and standard deviation of PRBC.

PRBC = packs of red blood cells; SD = standard deviation; CABG = coronary artery bypass graft.

The overall mortality in the hospital was 1.7% and postoperative complications (morbidity) were 38.4%. 190 patients (5.7%) required additional surgery for bleeding, 33 (1.0%) experienced permanent stroke according to the classification of Ergin and colleagues [14] and 17 patients (0.5%) had sternal wound infection. Acute renal failure requiring dialysis occurred in 71 patients (2.1%). The duration of mechanical ventilation was 28.4 ± 97.7 hours and length of intensive care was 3.0 ± 5.4 days. Chest tube drainage volume in 24 hours was 323 ± 328 ml.

**Figure 1:**
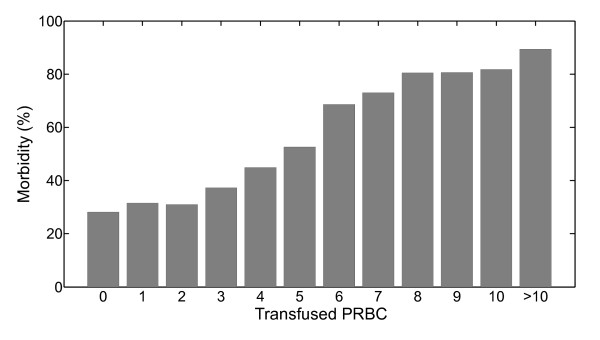
**Empirical distribution of percentage morbidity with respect to packs of red blood cells (PRBC) transfused**.

2208 patients (67%) were transfused. 710 patients (21%) received fresh frozen plasma, and 235 (7%) received platelets. Some patients received more than one type of blood product. Figure 1, showing percentage morbidity against number of PRBC transfused, highlights a clear increase in percentage morbidity with number of packs.

Table 4 shows the optimal subset of regressors obtained step-by-step by the variable selection procedure. Ten dummy variables were entered in the model and none of them were removed. After rounding off regression coefficients to the nearest half integer, dummy regressors equal to one corresponded to an integer number of half blood packs, as shown in the last column of Table 4.

**Table 4:** Stepwise procedure for dummy variable selection

**Step no**.	Dummy variables	Coefficients	Estimated PRBC
1	Hct_Admission _≤ 40%	0.928	1

2	CBP time > 130 min	0.951	1

3	Minimum Hct_CBP _≤ 20%	2.000	2

4	Surgical procedure different from isolated CABG	0.936	1

5	Age > 70 years	0.616	0.5

6	Cardiogenic shock	2.627	2.5

7	Preoperative dialysis	4.246	4

8	Systemic arterial hypertension	0.384	0.5

9	Urgency	0.692	0.5

10	Emergency	1.390	1.5

Regression model coefficients are transformed into packs of red blood cells (PRBC) by rounding them to the nearest half integer.

Hct = hematocrit; CBP = cardiopulmonary bypass; CABG = coronary artery bypass graft.

The RMSE on training data was less than three PRBC (2.76 PRBC). Testing data gave a RMSE = 2.81 PRBC. No statistical differences were found between training and testing sets for all model selected dummy variables (Fisher exact test, p > 0.05).

Comparing the actual numbers of packs transfused with model-estimated numbers, the following three groups of patients were defined on the basis of the estimated RMSE value (about 2.8 PRBC):

- Group I, where the difference between the model estimate and the actual number of packs transfused was greater than two (patients in group I were named "less transfused", because they received appreciably fewer transfusions than estimated by the model);

-  Group II, where the absolute value of the difference between the number of packs transfused and the number estimated by model was not more than two packs ("well transfused" patients);

-  Group III, where the difference between the actual number of packs transfused and the model estimate was greater than two packs ("over transfused" patients).

Taken as a whole, we identified 303 (9.1%) less transfused patients (151 in the training set and 152 the testing set), 2574 (77.7%) well transfused patients (1278 and 1296, respectively) and 438 (13.2%) over transfused patients (228 and 210, respectively). No statistical differences were found in the frequencies of groups I, II and III between the training and testing sets (chi-square test, p = 0.648), so all patients (in the training and testing sets) were pooled for further analysis.

It can be underlined that the RMSE-based criterion identified the group II of well transfused patients as positively consistent with a clinically acceptable model error. The percentage of over and less transfused patients was 22.3%. The number of packs of red blood cells was 0.53 ± 0.94, 1.7 ± 1.7 and 8.3 ± 4.7 for patients of groups I, II and III, respectively.

Percentage morbidity was rather different in the three groups. Group II showed the lowest morbidity (31.5%) and group III the highest (71.0%). An intermediate value (49.5%) was recorded in group I.

**Figure 2:**
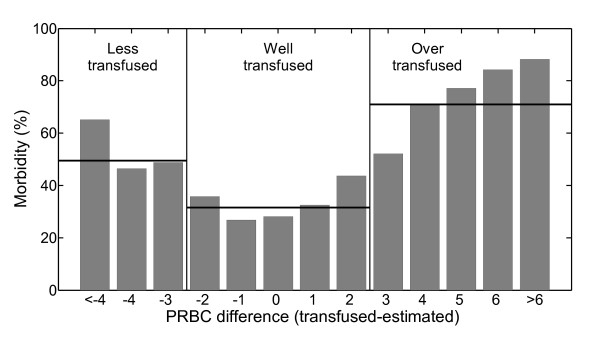
**Empirical distribution of percentage morbidity with respect to difference between actually transfused and model-estimated numbers of packs of red blood cells (PRBC)**.

Figure 2 shows percentage morbidity against the difference between the actually transfused and model-estimated number of PRBC. A negative (positive) difference meant that the model estimated more (fewer) PRBC than actually transfused. Vertical lines divide our sample and horizontal lines indicate the corresponding percentage morbidity in the three groups. Figure 2 highlights the remarkable increase in morbidity in less-transfused and over-transfused patients. A clear positive relationship is also evident between morbidity and difference between model-estimated and actual number of packs transfused. Percentage morbidity was about 28% in patients for whom the model fitted the actual data exactly, while the highest differences were associated with very high morbidities. In particular, percentage morbidity was about 65% with an absolute difference of more than four PRBC in group I patients ("less transfused"), and 88% with a difference of more than six packs in group III ("over transfused").

Statistical comparison of overall percentage morbidities by the chi-square test and Fisher exact test revealed significant differences between groups I, II and III. In particular, percentage morbidity in group I was significantly lower than in group III and higher than in group II.

Statistically significant differences in number of packs actually transfused were found between normal and morbid patients (Mann-Whitney test). On average, the number of packs transfused into morbid patients was more than twice that of normal patients (3.7 ± 4.3 vs. 1.7 ± 2.0 packs). The dummy-variable model estimated that morbid patients needed significantly fewer packs (2.7 ± 1.6 packs). For these patients the Wilcoxon test for paired samples demonstrated significant differences between model-estimated and actual number of packs transfused.

Closer analysis of cases belonging to groups I and III showed that:

- transfusion therapy not in line with our broad-based blood conservation strategy was prescribed in about 60% (182 out of 303) of patients in group I;

- about 82% (358 out of 438) of patients in group III had occasional, unpredictable, adverse events determined directly by surgery, such as bleeding, by-pass graft occlusion, heart failure requiring mechanical support, infection, coma or acute kidney failure.

Table 5 summarizes the descriptive statistics of all dichotomous predictors (preoperative and intraoperative features) included in the model and some essential postoperative variables for the three groups. The association between each predictive or postoperative variable and patient group was investigated analysing the corresponding contingency table. The chi-square test for independence showed statistically significant differences in all circumstances, except urgency. Concentrating our attention on postoperative issues, this indicated a strong association between the postoperative variables analysed and the three groups.

**Table 5:** Descriptive statistics for all dichotomous (preoperative and intraoperative) predictors included in the model and for some essential postoperative variables in three groups of patients: group I, where the difference between the model estimate and the actual number of packs transfused was greater than two (303 patients); group II, where the absolute value of the difference between the number of packs transfused and the number estimated by model was not more than two packs (2574 patients); group III, where the difference between the actual number of packs transfused and the model estimate was greater than two packs (438 patients)

Variables	Frequency count and percentage in group I	Frequency count and percentage in group II	Frequency count and percentage in group III
*Preoperative and intraoperative predictors included in the model*			

Hct_admission _≤ 40%	257 (84.8%)	1450 (56.3%)	266 (60.7%)

CBP time > 130 min	222 (73.3%)	929 (36.1%)	200 (45.7%)

Minimum Hct_CBP _≤ 20%	108 (35.6%)	333 (12.9%)	92 (21.0%)

Surgical procedure different from isolated CABG	230 (75.9%)	1098 (42.7%)	231 (52.7%)

Age > 70 years	212 (70.0%)	1146 (44.5%)	241 (55.0%)

Cardiogenic shock	9 (29.0%)	17 (54.8%)	5 (16.1%)

Preoperative dialysis	7 (2.3%)	8 (0.3%)	6 (1.4%)

Systemic arterial hypertension	243 (80.2%)	1701 (66.1%)	28 (64.6%)

Urgency	22 (7.3%)	220 (8.5%)	4 (10.3%)

Emergency	19 (6.3%)	37 (1.4%)	10 (2.3%)

*Postoperative variables*			

Morbidity	150 (49.5%)	812 (31.5%)	311 (71.0%)

Reoperation for bleeding	14 (4.6%)	57 (2.2%)	118 (26.9%)

Lung dysfunction	58 (19.1%)	280 (10.9%)	178 (40.6%)

Low cardiac output	54 (17.8%)	239 (9.3%)	149 (34.0%)

Cardiac arrhythmia	37 (12.2%)	193 (7.5%)	64 (14.6%)

Coma	2 (0.7%)	15 (0.6%)	18 (4.1%)

Stroke	3 (1.0%)	19 (0.7%)	11 (2.5%)

Acute kidney failure	4 (1.3%)	26 (1.0%)	41 (9.4%)

Kidney dysfunction	16 (5.3%)	103 (4.0%)	21 (4.8%)

Sepsis	2 (0.7%)	7 (0.3%)	17 (3.8%)

Pneumonia	3 (1.0%)	17 (0.7%)	31 (7.8%)

Sternal wound infection	2 (0.7%)	7 (0.3%)	8 (1.8%)

Death	4 (1.3%)	26 (1.0%)	28 (6.4%)

Mechanical ventilation > 1 day	50 (16.5%)	206 (8.0%)	166 (37.9%)

Intensive care > 5 days	30 (5.2%)	135 (9.9%)	128 (29.2%)

Hct = hematocrit; CPB = cardiopulmonary bypass; CABG = coronary artery bypass graft.

## Discussion

Heart surgery makes a large demand on available blood. It has been estimated that 11% of blood resources are used for transfusion support in patients undergoing CABG [2] and nearly 20% of blood transfusions are related to heart surgery [15]. Despite major advances in perioperative blood conservation, the transfusion rate in heart surgery patients remains high and large differences can be observed in different centres [16]. In the present study we found an overall transfusion percentage of about 67%, only partially justified by the large number of emergency and redo operations or high risk procedures requiring prolonged CPB time.

Transfusion therapy, although recognised to be necessary during heart surgery, may lead to an increased incidence of complications. In fact, despite the difficulty of separating the effects of transfusions from the underlying severity of the clinical condition, several prior studies have demonstrated that allogeneic blood transfusions are associated with an increased morbidity rate (e.g. atrial fibrillation, renal dysfunction, pulmonary complications and low cardiac output syndrome) [17-20] and worse long-term survival [21]. Recently, Koch and colleagues also showed that the length of storage of PRBC is associated with increased morbidity and mortality in heart surgery and that transfusions with blood stored for more than 14 days are associated with a significantly increased in-hospital mortality rate [22]. This evidence has stimulated interest in developing different strategies of blood transfusion and conservation in heart surgery and in identifying patients really requiring transfusions during and/or after surgery.

Several authors analysed critical aspects of blood transfusions in terms of morbidity and mortality and used preoperative predictors for allogeneic transfusions in order to develop a predictive transfusion risk score for patients undergoing heart surgery [8,9,11]. Magovern and colleagues analysed a sample of patients undergoing isolated CABG procedures including emergency cases and reoperations [8]. They observed that 61% received transfusion during hospitalization, developing a model, which predicted the need for transfusion after CABG, based on 14 preoperative variables as predictors. Similarly, Alghamdi and colleagues developed a model for predicting the need for blood transfusion based on eight preoperative variables [9]. Ranucci and colleagues developed a predictive score using five predictors extracted as the most clinically relevant based on the judgement of 30 clinicians concerned with transfusions in heart surgery [11]. Although built on a subjective choice of a few predictors, the score seemed to have suitable predictive power and calibration.

A general weakness of the above studies could be the use of only preoperative predictors. Moskowitz and colleagues proposed six preoperative predictors and four intraoperative predictors to create a formula to predict transfusion requirements for major heart surgery procedures in a centre that implements a multimodal approach to blood conservation. However, they limited analysis to a sample of only 307 consecutive patients undergoing CABG, valve, and combined procedures (CABC plus valve), where only 35 patients required intraoperative or postoperative allogeneic transfusions.

In the present analysis, we selected a set of preoperative and intraoperative variables statistically to develop a dummy-variable linear-regression model using a sample of 3315 consecutive heart surgery patients, where the size of transfused and not-transfused samples was sufficiently large. The role of dummies is to partition the data set into groups based on qualitative criteria: researchers in economics and the social sciences make wide use of linear regression models in which the dependent variable is continuous-valued while the regressors are dummy variables. Despite its recognized capabilities, this approach is not yet extensively used in medical studies and to our knowledge, no model for predicting the need for transfusions in heart surgery patients has been developed by this method.

The present model selected eight preoperative and two intraoperative dummy regressors, which in our experience are associated with need for blood transfusion (admission hematocrit ≤ 40%, CPB > 130 minutes, hematocrit at CBP ≤ 20%, operation different from isolated CABG, age > 70 years, cardiogenic shock, preoperative dialysis, systemic arterial hypertension, urgent and emergency operation). Approximating the regression coefficients to the nearest half unit, each dummy variable equal to 1 gave an integer number of half PRBC. Thus the model enabled prompt and simple planning of transfusions needs, patient-by-patient.

Most of the above variables were identified as important transfusion predictors in other studies. Hardy reported that the risk of exposure to blood transfusion increased in patients undergoing combined cardiac or valve procedures and that urgent surgery was a key independent predictor of exposure to blood transfusion [23]. In a systematic review study, different variables were found to be associated with increased red cell transfusion rates: these included renal insufficiency, urgent/emergency surgery, low hematocrit and older age [24]. In particular, a decline in kidney function (insufficiency or failure) was associated with significantly increased transfusion rates, because the odds of transfusion increased 1.5- to 8-fold. Similarly, urgent or emergency surgery was identified as a very important risk factor associated with a 4- to 8-fold increase in transfusion rates compared to elective surgery. The conclusion was that transfusion practices for adults having emergency or urgent surgery need to be optimized because of the effect of usually prescribed anticoagulant and antiplatelet agents on blood loss. The report from the STS Workforce on Evidence Based Surgery indicates that numerous studies have identified prolonged CPB time as a risk factor associated with increased transfusions [2], in line with increased blood cell damage and coagulation disorders due to alteration of the coagulation cascade [25]. Swaminathan and colleagues found an association between lowest hematocrit during CPB and increased transfusion [26], while Takami and Masumoto recently indicated that independent factors for allogeneic blood transfusion included preoperative and minimum hemoglobin values during cardiopulmonary bypass [27]. The association between low preoperative hematocrit and increased transfusion is generally confirmed by several studies [2].

The use of dummy variables in linear regression models is very useful when the data set has to be partitioned into groups based on qualitative criteria, but the inclusion of dummies tends to degrade the robustness of linear regression estimators when the sample contains anomalous observations. In the present paper we critically reviewed the transfusion strategy of all patients to test the model from a clinical point of view. In particular, we identified three groups of patients analyzing the difference between the model-estimated and actual number of packs transfused. On the basis of the model RMSE we defined a group of "less transfused" patients (Group I, 9.1%) who received appreciably fewer packs than estimated by the model. Similarly, the group of "over-transfused" patients (Group III, 13.2%) included all patients who received appreciably more blood packs than estimated by the model. Of course, the group II contained the majority patients (77.7%) who showed less difference between estimated and actual number of packs. *A posteriori *analysis showed that during surgery, about 82% of patients in group III had an unforeseeable adverse event such as bleeding, by-pass graft occlusion, heart failure requiring mechanical support, infection, coma or acute kidney failure, while about 60% of patients in group I received transfusion therapy at variance with our broad-based blood conservation strategy. When these patients were removed from the sample, the percentage of cases in which the model estimate and the actual number of packs transfused differed by more than two packs, decreased to only 7%. This suggests that model outcome is clinically reliable and model errors are sufficiently small.

We found significantly higher morbidity in patients of groups I and III. Surprisingly, *a posteriori *analysis of EUROSCORE (not reported in Results), did not reveal any difference in EUROSCORE [28] between the three groups. It is interesting that the increase in morbidity was much more evident in group III where it reached 71.0% against 31.5% in group II. In line with several previous studies, this seems to suggest that "unnecessary" transfusions may be associated with an increase in complications. Although the model estimated less need for transfusion in patients of group III, the actual model capability in these critical cases needs to be evaluated in more detail. The present retrospective analysis did not allow us to clarify this controversial matter once and for all. The problem could perhaps be better analysed by a further prospective study, comparing outcomes in randomized trials. However, this is a not simple point, because instead of causing complications, transfusions are often given when there are heart surgery complications and this association may reflect a tendency to transfuse critically ill patients. In any case, the model is an aid to clinical decision making, providing quantitative information and alerting medical teams to reconsider decisions diverging radically from model prediction.

The present model does not predict unforeseeable adverse events during surgery and therefore cannot forecast the actual need for transfusion in patients with such problems. However, it can be a useful auxiliary to plan transfusion needs *a priori *in most situations, enabling a reduction in healthcare costs.

In short, our study underlines the need for standardization in transfusion practices and suggests a very simple transfusion model that can facilitate optimization of administration of blood products.

## Conclusions

Unnecessary blood transfusions during heart surgery increase healthcare costs directly, because blood is an increasingly scarce and expensive resource, and indirectly, due to complications associated with transfusions. Clinical use of a simple and reliable transfusion model can improve conservation strategy and optimize administration of blood products.

In the patient sample studied, a dummy-variable linear-regression model proved to be a convenient tool for predicting transfusion needs *a priori *in most heart surgery situations on the basis of some preoperative and intraoperative information. Clinical use of this type of model is extremely simple and immediate, since each dummy predictor equal to one directly indicates an estimated number of blood packs.

In conclusion, although further validation is necessary, the results clearly indicate that the present modelling approach enables design of a useful decision system for planning transfusion needs in patients after heart surgery.

## Abbreviations

TRUST: transfusion risk understanding scoring tool; TRACK: transfusion risk and clinical; CABG: coronary artery bypass grafting; CPB: cardiopulmonary bypass; PRBC: packs of red blood cells.

## Competing interests

The authors declare that they have no competing interests.

## Authors' contributions

All authors participated in the study plan and coordination. FS collected clinical data. FS, FF, AM, VF, SS, BB and PG were concerned with all clinical aspects of the study. GC and PB designed the model and performed data processing and statistical analysis. All authors read and approved the final manuscript.

## Pre-publication history

The pre-publication history for this paper can be accessed here:

http://www.biomedcentral.com/1472-6947/11/44/prepub
